# Pregnancy-related chronic type A aortic dissection highlights the importance of thorough prenatal maternal examination

**DOI:** 10.1186/s13019-025-03357-2

**Published:** 2025-01-29

**Authors:** Cristina M. Șulea, Anna B. Kiss, Bence Ágg, Kálmán Benke, Elektra Bartha, Bálint Szilveszter, Roland Stengl, Máté Csonka, Zoltán Szabolcs, Miklós Pólos

**Affiliations:** 1 Semmelweis University Heart and Vascular Centre, Budapest, 1122 Hungary; 2 Hungarian Marfan Foundation, 1122 Budapest, Hungary; 3 Center for Pharmacology and Drug Research & Development, Department of Pharmacology and Pharmacotherapy, Semmelweis University, 1089 Budapest, Hungary

**Keywords:** Aortic dissection, Pregnancy, Disease prevention

## Abstract

**Background:**

Aortic dissection occurs rarely during pregnancy but carries a significantly high vital risk for both the mother and the fetus. Early diagnosis and treatment are critical for a successful outcome.

**Case presentation:**

A 32-year-old pregnant woman at 31 weeks of gestation began experiencing shortness of breath, chest pain, and palpitations, which were attributed to an anxiety disorder she had been previously diagnosed with. The symptoms continued to worsen following the delivery when a chest computed tomography investigation revealed signs of chronic type A aortic dissection and an 80 mm post-dissection aneurysm of the ascending aorta. Aortic repair via composite aortic root replacement surgery (Bentall procedure) and partial aortic arch replacement was performed. The patient’s postoperative evolution was notable for a series of complications.

**Conclusions:**

Our report highlights the importance of thorough maternal examination during pregnancy. The high mortality rates associated with aortic dissection occurring in pregnant women and the possibility of missed intervention due to atypical clinical presentation warrant the need for standardized international protocols aimed at the prevention and timely diagnosis of prenatal aortic disease.

## Background

Aortic dissection (AD) is a rare but potentially life-threatening cardiovascular condition that can occur both during pregnancy and in the postpartum period, with an estimated rate of 5.5 per 1 million cases [1]. Aortic rupture is one of its fatal complications. Although the aortic wall can temporarily bear the increased blood pressure, the strain may cause it to weaken and become permeable, thereby leading to the development of cardiac tamponade, hemothorax, retroperitoneal or peritoneal bleeding. Mortality is exceedingly high in acute Stanford type A AD cases, rising by 1–2% per hour immediately after the onset of symptoms [2]. Type A cases account for 67% of all ADs occurring during pregnancy [3]. AD-related maternal mortality is estimated to be around 23%, whereas fetal mortality is slightly higher, at approximately 27% [3]. Considering the significant vital risk, immediate operative intervention is essential for an improved prognosis. In surgically treated cases, the 30- and 90-day mortality rates are around 15% and 19%, respectively, compared to 62% and 67% for non-surgically treated patients. The survival rate inversely correlates with the maximum diameter of the aorta, a more favorable outlook being observed at smaller aortic measurements [4].

Pregnancy is associated with a significantly increased risk for AD and aortic rupture compared to non-pregnant women. 50% of ADs in female patients under the age of 40 develop during pregnancy. These cases account for 3–10% of maternal mortality [5]. Nevertheless, the occurrence of AD in pregnancy is intricately linked to underlying genetic and anatomic predispositions. Women with connective tissue disorders (e.g. Marfan syndrome (MFS), Loeys-Dietz syndrome, Turner syndrome, vascular Ehler-Danlos syndrome) or other non-syndromic heritable thoracic aortic diseases (HTAD) are at higher risk during pregnancy [6]. More than 50% of the cases occur in women affected by MFS. The incidence of AD during pregnancy in this population is 3% [5]. Dissection occurs much earlier in women with MFS than in previously healthy women. The risk is also up to eighteen times higher in gravidas who have a bicuspid aortic valve instead of a tricuspid one [7]. Bicuspid aortic valve has a prevalence of 1–2% in the total population and is responsible for 3–7% of ADs [5]. When it is accompanied by a dilated aortic root, the risk becomes similar to that of patients with MFS [7]. Pre−existing or pregnancy−induced hypertension (preeclampsia) further increase the risk of pregnancy−related AD by up to threefold [1].

Due to the increased cardiovascular stress caused by pregnancy, the risk also rises with advancing gestational age. Most cases of AD occur in the third trimester and in the early postpartum period, less often in the second, and even less commonly in the first trimester. In women without any connective tissue disorder, AD only develops during the third trimester and the postpartum period [7]. Evidence suggests that cardiac output increases during pregnancy and rises above non−pregnant levels as early as the first trimester. It reaches its peak in the first part of the third trimester and remains higher during labor and also immediately after delivery [8]. Uterine contractions significantly increase the cardiac output, probably due to the 300–500 ml autotransfusion of maternal blood and the increase in heart rate. This transient autotransfusion during contractions also causes a 10 mmHg rise in central venous pressure, mean arterial pressure, and pulmonary wedge pressure as well. Finally, in the second phase of labor, the Valsalva maneuver during the pushing phase and the effort exerted during pushing pains are associated with a significant, short−term increase in maternal systemic arterial pressure. In addition to traditional cardiovascular risk factors, pregnancy−specific risk factors for AD include gestational diabetes and hypertension, eclampsia, preeclampsia, multiple pregnancy, and older maternal age. Gestational diabetes is more common in postpartum dissections [9].

Women with a history of a cardiac event, stage III or IV heart failure based on the New York Heart Association Classification, or left ventricular obstruction, have a sixfold greater risk of an adverse cardiac event during pregnancy than women without these factors. Among pregnant women with systolic dysfunction, this occurrence is eleven times more common than in women with normal ventricular function [10]. In many cases, the existence of maternal risk factors remains unknown and is revealed only when the dissection occurs. Assessing the risk of pregnancy, preventing complications, and carrying out any necessary prophylactic interventions are essential for the safety of both mother and fetus. This may be achieved only through extensive monitoring before and during pregnancy, which would facilitate the discovery of risk factors in time, thus effectively reducing the frequency of cardiovascular disease [11].

## Case presentation

A multiparous 32-year-old postpartum female patient was transferred to our center for further investigation on suspicion of aortic disease. Six days prior to admission, she had given birth to her third baby per vias naturales at 41 weeks. She had a past medical history of anxiety disorder and regular use of anti-anxiety medications. The diagnosis of MFS was ruled out based on physical examination, medical and family history. Following an initially uneventful development of the pregnancy, at the end of the second trimester, the patient reported experiencing shortness of breath, chest pain, and palpitations. After the delivery, her symptoms worsened, and she was recommended psychological support in the psychiatric ward and low-dose benzodiazepine therapy on account of postpartum anxiety. Less than a week later, she suffered three consecutive panic attacks and developed chest pain, dyspnea, headache, and sweating. Arterial blood gas analysis showed hypocapnia and base deficit (pCO_2_, 28 mmHg; HCO_3_^-^, 18.5 mmol/L). A presumptive diagnosis of pulmonary embolism was made, and the patient underwent a thoracic computer tomography (CT) angiography, which uncovered pronounced dilatation of the ascending aorta dislocating the right pulmonary artery and the superior vena cava, cardiomegaly, and congestion.

**Fig. 1:**
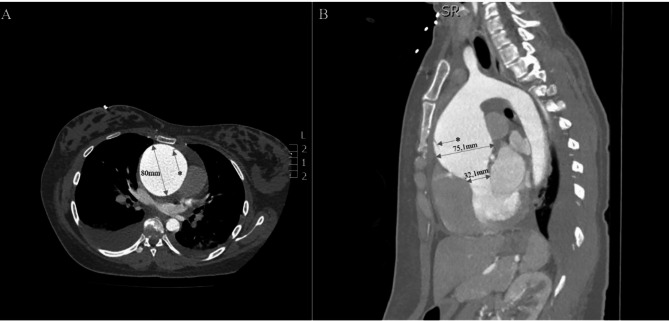
Preoperative CT angiography axial **(A)** and parasagittal **(B)** views depicting the intraluminal intimal flaps (marked with an asterisk) and the significant size of the post-dissection aneurysm of the ascending aorta

In our center, a further acute CT angiography was performed, and the picture was suggestive of chronic Stanford type A AD with a significant (~ 80 × 75 mm) ascending aortic aneurysm (Fig. 1). The presence of an intraluminal intimal flap, or true lumen/false lumen separation, was not clearly depicted. There was little pericardial and moderate bilateral pleural fluid, with right-sided predominance. Transthoracic echocardiography (TTE) identified a bicuspid aortic valve.

**Fig. 2:**
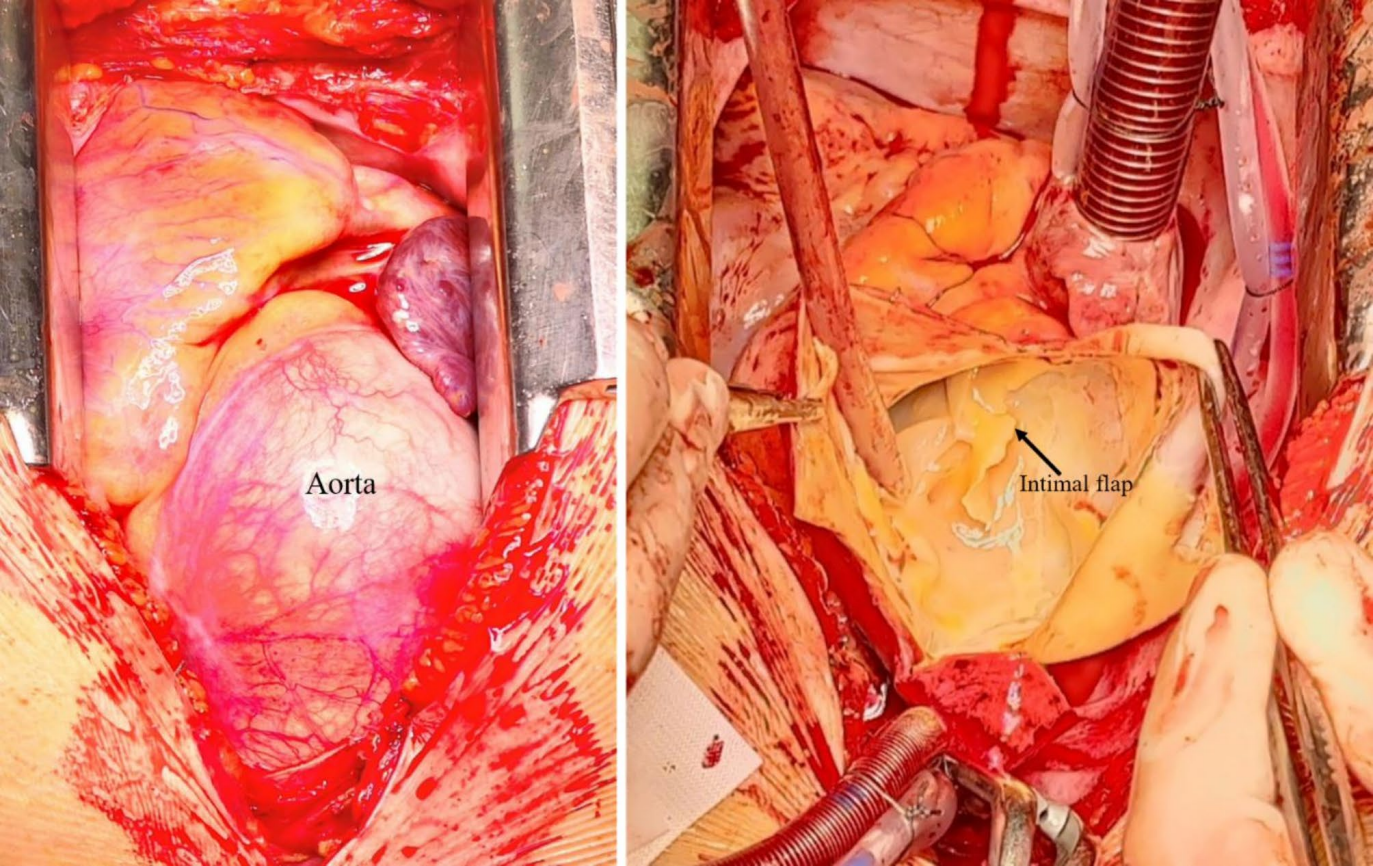
Intraoperative view of the enlarged aortic root. The intimal flaps were clearly visible upon the opening of the aorta

Following preoperative preparation with levosimendan due to decreased left ventricular ejection fraction (35%), a Bentall operation with partial aortic arch replacement was performed the next day (Fig. 2). A vascular conduit equipped with a 25 mm mechanical valve (St. Jude Medical, Minneapolis, Minnesota, USA) was implanted. Partial aortic arch replacement was performed in deep hypothermic (18 °C) circulatory arrest (12 min). The patient was transferred to the intensive care unit with satisfactory hemostasis and no intraoperative complications.

On the intensive care unit, optimal medical therapy was administered over the course of one week. The patient was then transferred to the cardiac surgery ward, where she received treatment for dilated cardiomyopathy and underwent further postoperative management. Lactation was suspended with bromocriptine, which was later gradually abandoned by the patient due to side effects. Additionally, benzodiazepine-induced anxiolysis was used. Control TTE investigations showed a gradual amelioration in left ventricular function, the ejection fraction reaching 40% by the 14th postoperative day.

**Fig. 3:**
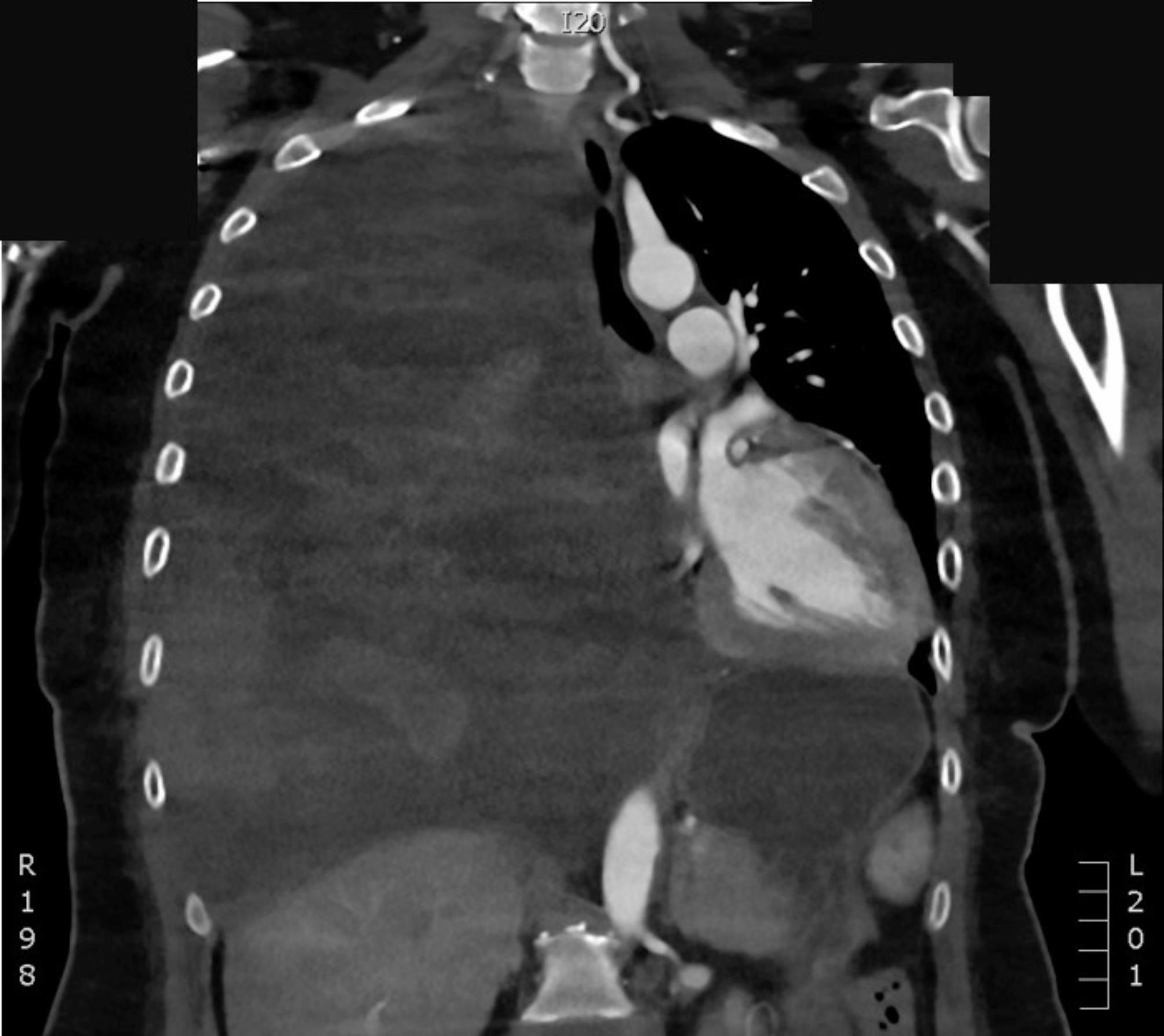
CT angiogram showing a massive right-sided thoracic hematoma that was dislodging the mediastinum. The effusion was compressing the pulmonary arteries and the superior vena cava

At 15 days postoperatively, due to generalized weakness, fatigue, and low blood count (hematocrit, 18%; red blood cell count, 2.01 T/L; hemoglobin, 6.1 g/dL), an acute CT angiography of the chest, abdomen, and pelvis was performed, revealing an active retrosternal bleeding at the level of the aortic arch and significant right-sided thoracic effusion (Fig. 3). Via median re-sternotomy, a volume of roughly 3000 ml of blood (both fresh and coagulated) was evacuated from the pericardial sac.

After two days of observation on the intensive care unit, the patient was mobilized, and breathing exercises were initiated. Due to further hypotension and anemia, she received 2 units of red blood cell transfusion and intravenous fluid replacement. Her symptoms prompted another CT angiography scan of the chest, abdomen, and pelvis, which uncovered a 4 cm hematoma at the upper edge of the oblique fissure, but no other signs of active bleeding. Therefore, no further surgical intervention was deemed necessary.

The patient was discharged in stable condition without complaints on the 32nd postoperative day, by which control TTE showed a steady improvement in left ventricular function (ejection fraction of 45%). Further control TTEs performed at 6 and 12 months postoperatively showed amelioration in left ventricular function up to a physiological ejection fraction of 60%.

## Discussion and conclusions

The rate of AD is estimated to be around 0.0004% during pregnancy [12]. Despite known predisposing conditions, mainly a history of HTAD, it has been previously reported to occur even in patients without risk factors [1]. Literature holds relatively few pregnancy-related AD cases, out of which non-syndromic, sporadic cases, such as the one presented, represent the minority [13, 14]. Despite the differences in timing and clinical presentation, all cases highlight the importance of a high degree of clinical suspicion for AD and thorough physical and non-invasive maternal examinations during pregnancy, even in women with no relevant history or risk factors. Here we presented a case of pregnancy-related AD developed conceivably on the basis of underlying aortic valve disease.

After confirming the existence of pregnancy, pregnant women must undergo thorough internal medicine consultations. Currently, in Hungary, their cardiovascular condition is assessed by blood pressure and heart rate measurements and an electrocardiography examination during the first trimester, and then blood pressure and heart rate measurements are performed at each examination during the next two trimesters of pregnancy. The recommendation is similar in the United Kingdom [15], whereas, in the United States of America, only blood pressure and heart rate measurements are prescribed [16]. These parameters can draw attention to the existence and/or development of certain cardiac disorders that existed before or occurred during pregnancy (e.g. hypertension, arrhythmias), but they are not sufficient for the detection of numerous other possible cardiovascular conditions.

The routine physical examination allows for performing stethoscopic auscultation. Although the diagnostic potential of this method is limited, in certain situations, such as the one presented, the maneuver could hint at the existence of an aortic valve disease. Its availability and accuracy in detecting pathologic heart murmurs, even in asymptomatic patients, make cardiac auscultation an important screening tool in obstetric patients [17]. Similarly, a thorough examination of the symptomatology of pregnant women also deserves great emphasis [18]. If justified based on the existing signs and symptoms and the presence of risk factors, specialist referrals are of utmost importance during prenatal care as these professionals have access to advanced diagnostic tools and can provide advanced monitoring and interventions that are often beyond the scope of general practitioners or obstetricians. Regardless of the type of investigation, a comprehensive physical examination is crucial for differential diagnosis [19]. In this way, it is possible to avoid missed opportunities for intervention in the case of undiscovered diseases, as well as unnecessary drug treatments prescribed following a misdiagnosis. A supporting example is the present case, where the palpitations attributed to the patient’s anxiety and frequent panic attacks could have drawn attention to the existence of cardiovascular disease, not only during visits in the framework of prenatal care but also during her psychiatric treatment.

The reliability of the prenatal cardiovascular assessment may be further improved by performing echocardiography during pregnancy, as it is a non-invasive, painless, and radiation-free method, and has no harmful effects on the conceptus. Currently, TTE is recommended for all pregnant women with known cardiovascular disease or a history of exposure to cardiotoxic risk factors [20]. However, abnormal echocardiographic findings were found to be associated with adverse obstetric outcomes in pregnant women without known cardiac disease [21]. TTE may help in the detection and severity assessment of potential valvulopathies behind heart murmurs discovered during physical examination, some congenital heart malformations, but also in investigating the condition of the large blood vessels, the presence of wall-motion abnormalities, and the status of the myocardial pump function [22, 23]. Considering that pregnancy can deteriorate underlying cardiac diseases or precipitate de novo conditions, echocardiographic surveillance of pregnant women is crucial for improving maternal outcomes. Nonetheless, the method presents itself with certain difficulties related to pregnancy, due to which the accuracy of the method greatly depends on the examiner’s expertise [24]. These issues could be addressed by integrating cardiology examinations into prenatal care.

If AD is suspected in unstable patients, transesophageal echocardiography (TEE) is recommended. TEE provides high-resolution images of most of the thoracic aorta, apart from a short segment of the distal ascending aorta just proximal to the innominate artery, attributable to acoustic shadowing from the trachea [25]. It has very high sensitivity and specificity for diagnosing ADs and can be used to detect and investigate aortic aneurysms, hematomas, or thrombi [26, 27]. Despite the slightly elevated risk of aspiration attributed to the increased intra-abdominal pressure, TEE is considered safe during pregnancy [23]. It requires an experienced sonographer and sedation, but under suitable conditions, without the presence of ionizing radiation, it can be conveniently used to assess the condition of the aortic wall.

Preoperative imaging in the case of pregnancy involves additional risks. In aortic surgery, the ideal imaging procedure for diagnosis and preparation of the surgical plan is CT angiography, with which AD, intramural hematoma, and ulceration can be safely distinguished. Compared to the old gold standard aortography, it also has the advantage of non-invasiveness. Despite all this, performing a CT scan exposes both the mother and the fetus to radiation and carries certain dangers for the latter [28]. The availability of MRI is increasing in diagnostics, as it eliminates radiation and is considered safe for the fetus [29]. This method can be used to assess left ventricular function, aortic valve disorders, and the involvement of branching arteries. However, its disadvantage lies in the longer duration of the examination, which subjects the gravida to a prolonged supine position, in which the pregnant uterus can cause aortocaval compression [30, 31].

The sensitivity of the aforementioned imaging procedures (CT, MRI, TEE) for detecting AD is similar (93.8%, 98.3%, and 97.7%, respectively), and they are much more accurate than TTE (59.3%). Regarding their specificity, both TTE (83.0%) and TEE (76.9%) fall short of CT (87.1%) and MRI (97.8%) [32]. For TTE, a higher sensitivity was found in the diagnosis of ascending AD (78–90%) compared to descending AD (31–55%), while specificity for type A AD surpassed that for type B AD (87–96% and 60–83%, respectively) [33, 34]. CT and MRI are more sensitive than TTE for the detection of thrombus formation affecting the thoracic segment of the aorta, but in this regard, they do not perform better than TEE. CT is inadequate for detecting AD entry points and aortic valve regurgitation, but MRI and TEE can accurately identify these [32]. The optimal approach to the detection of AD during pregnancy is a non-invasive, radiation-free diagnostic strategy, with which the comprehensive evaluation can be reduced to a single non-invasive diagnostic test.

The maternal mortality rate for all heart surgeries during pregnancy has been reported to range from 3 to 11%, and it is highest (22%) for interventions for AD and pulmonary embolism, which reflects the severity of the mother’s condition at the moment of surgery [35]. The fetal and neonatal mortality rates associated with maternal heart surgery during pregnancy are even higher, ranging from 20 to 30% [36]. Moreover, the surgical reconstruction of the ascending aorta requires the use of cardiopulmonary bypass, which has been found to carry an even higher risk for the conceptus than the mother. Given the gestational age, different management options are available. If critical aortic dilatation or dissection is diagnosed during the first trimester, termination of pregnancy should be considered. Due to the extreme risks to the fetus, if the gravida is in the third trimester, great attention should be paid to cesarean delivery before the cardiac surgery intervention using cardiopulmonary bypass. At earlier gestational ages, when this is not feasible, modifications to the perfusion protocol should be considered, including higher flow rate, normothermic perfusion, pulsatile flow, and the use of intraoperative external fetal heart rate monitoring [35]. When using cardiopulmonary bypass, it is important to monitor maternal carbon dioxide and pH levels, as alkalosis can reduce blood flow to the uterus [37]. This avoids the increased load due to higher blood pressure, which occurs during births per vias naturales and puts pressure on the dilated aortic wall and threatens the risk of aortic rupture. Surgery can be performed with less risk after childbirth. Despite all these possibilities, the primary goal should be the prevention and early diagnosis of AD.

Finally, the postpartum management of women at risk of pregnancy-related AD requires particular considerations. Rigorous surveillance is essential during the first six to twelve postpartum weeks to monitor aortic integrity and identify potential complications, as most dissections related to pregnancy may occur throughout this period [38]. Pharmacological management, particularly with beta blockers, should be considered for pregnant women with MFS or non-syndromic HTAD to control blood pressure and minimize aortic wall stress [39], despite their known effect to impair fetal growth [40, 41]. While breastfeeding is generally encouraged, its implications in this context should be carefully considered, with providers counseling patients on potential risks associated with oxytocin-induced vascular changes as suggested by animal studies [42, 43].

In conclusion, the dissection of the aorta during pregnancy is a very rare condition, especially among women who do not suffer from connective tissue disorders. Therefore, the correct diagnosis is often made late. Given the serious complications and high maternal and fetal mortality rates associated with AD during pregnancy and the early postpartum period, a thorough cardiovascular examination during pregnancy is vital. It is important to rule out risk factors in predisposed patients and establish a correct diagnosis in atypical cases, especially if suggestive symptoms occur. For this, it is necessary to carry out careful physical examinations during prenatal care and, in case of suspicion, to initiate further investigations. Through this, the discovery of not only AD but also other cardiovascular pathologies can become more feasible, making pregnancy safer.

## Data Availability

No datasets were generated or analysed during the current study.

## Abbreviations

AD: Aortic dissection
CT: Computed tomography
HTAD: Heritable thoracic aortic disease
MFS: Marfan syndrome
MRI: Magnetic resonance imaging
TEE: Transesophageal echocardiography
TTE: Transthoracic echocardiography
